# Perceptions of challenges in clinical pediatric research: a survey of Italian pediatricians

**DOI:** 10.1186/s13052-026-02282-x

**Published:** 2026-06-12

**Authors:** Alberto Eugenio Tozzi, Ileana Croci, Claudio Pignata, Rino Agostiniani, Viviana Moschese

**Affiliations:** 1 Predictive and Preventive Medicine Research Unit, Bambino Gesù Children’s Hospital, IRCCS, Piazza S. Onofrio, 4, 00165 Rome, Italy; 2 Department of Translational Medical Sciences, Pediatric Section, Federico II University, Naples, Italy; 3 Department of Pediatrics, San Jacopo Hospital, Pistoia, Italy; 4 Pediatric Immunopathology and Allergology Unit, Policlinico Tor Vergata, University of Rome Tor Vergata, 00133 Rome, Italy

**Keywords:** Pediatric research, Clinical research barriers, Scientific publishing, Research evaluation metrics, Scientific writing, Research training

## Abstract

**Background:**

Clinical research is essential for advancing pediatric care, yet pediatricians often face unique structural, organizational, and methodological barriers that may hinder their participation in scientific activities. Understanding these obstacles is crucial to designing effective strategies that strengthen pediatric research capacity in Italy, a country with a leading role in European pediatric output. This study aimed to investigate Italian pediatricians’ perceptions of barriers, facilitators, and needs related to conducting research, applying evidence in clinical practice, and publishing scientific work.

**Methods:**

We conducted a cross-sectional online survey of Italian pediatricians using an 18-item questionnaire administered between April and May 2025. Data were analyzed descriptively, and multilevel logistic regression assessed associations between perceived barriers and respondents’ characteristics.

**Results:**

A total of 717 pediatricians involved in clinical and/or research activities were included. Among those engaged in research (*n* = 385), the most frequently reported personal barriers were difficulties balancing clinical and research duties (79.3%) and limited access to funding (67.9%). Operational issues such as delayed Ethics Committee approvals (80.8%) and challenges in multicenter data sharing (55.9%) were also prominent. Many respondents expressed dissatisfaction with current research evaluation systems: 78.2% believed that metrics should reflect long-term patient impact, and 73.1% reported that existing criteria penalize pediatric researchers in low-resource settings. Regarding scientific publishing, major obstacles included limited time (75%), complexity of data analysis (58.7%), and publication costs (55%). Interest in support initiatives was high, particularly for monthly evidence summaries (88.5%) and training in artificial intelligence tools (74.3%). Multivariate analysis revealed significant differences by age, gender, and professional role, especially concerning perceived research training adequacy and data analysis difficulties.

**Conclusions:**

Italian pediatricians face substantial structural, organizational, and methodological barriers to conducting research. Despite this, they show strong motivation to engage in scientific activities and express clear needs for training, infrastructural support, and improved evaluation systems. Strengthening funding opportunities, enhancing institutional support, and streamlining ethical and administrative procedures are essential to advancing pediatric research capacity and improving future child health outcomes.

**Supplementary Information:**

The online version contains supplementary material available at 10.1186/s13052-026-02282-x.

## Introduction

Conducting clinical research studies requires a competitive search for funding, appropriate infrastructures for research, and participation in research networks. Clinical researchers operate at the complex intersection of scientific rigor, patient advocacy, and regulatory compliance. One of the cornerstones of scientific research is knowledge sharing, which aims to multiply the impact of novel discoveries and that is mainly achieved through peer reviewed, scientific publications. Monitoring scientific production is achieved through several indicators, including the impact factor and the number of citations of published articles [1]. Since these indicators may be used for ranking individual researchers and institutions, the publish or perish imperative affects professional careers, incentives, and access to research funds [2]. However, clinical researchers struggle to allocate adequate time for this activity due to their demanding clinical responsibilities. This may add to other challenges in conducting clinical research such as insufficient funding, significant operational challenges [3], bureaucracy and regulation.

Investing in pediatric research is a direct investment in the future of society [4]. The progress achieved through dedicated scientific inquiry into childhood diseases has profoundly improved the quality of life for countless children, setting the foundation for healthier adulthoods. Given that pediatric research constitutes a small portion of the overall research landscape, certain challenges are significantly amplified in this specific field: access to research funding may be more difficult than in research fields for adults, and metrics for the evaluation of publications in peer reviewed journals may be less rewarding [3]. Nevertheless, strategies for promoting efficiency in pediatric scientific research and consequently publishing, are needed to constantly generate solutions that may translate into a better quality of care for children.

Italy plays a leading role in pediatric research in Europe [5] and scientific publications in this discipline are flourishing. While this is a supportive finding, maintaining and improving this positive trend requires further strategies [6, 7]. We therefore conducted a descriptive survey targeting the entire population of Italian pediatricians, with the aim of investigating the following areas: (1) what are the barriers and facilitators of pediatric clinical research; (2) how pediatricians translate evidence from scientific literature into clinical practice; (3) what are challenges encountered as authors when publishing scientific papers; and (4) what are potential actions to support pediatric clinical research.

## Methods

### Study design and participants

We conducted a cross-sectional survey among Italian pediatricians including physicians engaged in pediatric clinical practice and/or research activities. We invited Italian pediatricians through the communication channels of the Italian Society of Pediatrics that includes nearly 11.000 registrars, including newsletters and social media announcements. We administered a questionnaire through the SurveyMonkey platform (San Mateo, CA, USA). The survey was available for completion from April 15 to May 30, 2025.The present study consisted of an anonymous online survey administered to adult participants. No identifiable or sensitive personal data were collected, and participation was entirely voluntary. Participants were informed about the purpose of the study, consent was implied through survey completion, and withdrawal was possible while compiling the questionnaire. For these reasons ethical committee approval was not sought. All procedures performed in this study were consistent with data protection regulations (GDPR).

### Sample size

The sample size was estimated using the Raosoft software. Assuming an expected proportion of 50% for the outcomes of interest, with a 5% margin of error and a 95% confidence level, the minimum required sample was 372 participants.

### Questionnaire development

A literature review was conducted to identify existing instruments addressing similar topics. Although several studies explored aspects of clinical research behavior in different contexts, no previously validated questionnaire was found that comprehensively covered all the domains relevant to our objectives. Therefore, the authors developed a draft questionnaire from scratch, which was iteratively refined through structured discussions among the research team. The questionnaire used in this study was developed ad hoc with a primarily descriptive purpose, in alignment with the specific objectives of the study. It consisted of 18 items organized into five sections. The authors, who have expertise in pediatric research and clinical investigation, identified four key domains to describe the national landscape of scientific research: (1) barriers and facilitators of clinical research; (2) implementation of evidence from scientific publications; (3) authoring scientific papers; and (4) needs and support in clinical research.

To enhance clarity, relevance, and comprehensibility, the draft was pilot-tested with five individuals, including both researchers and individuals not routinely involved in pediatric research. Their feedback contributed to improving the wording and content of the items. Following this process, the questionnaire underwent final revision and was approved by all authors. As the questionnaire was purely descriptive, it did not undergo formal validation (Additional file 1).

### Statistical analysis

Categorical variables were summarized as frequencies and percentages. Univariate differences were assessed using Pearson’s Chi square test. All questionnaire items were measured on a 5-point Likert scale ranging from “strongly disagree” to “strongly agree”. For multivariable analyses, responses were dichotomized a priori: “agree” and “strongly agree” were considered as indicating the presence of the perceived barrier or difficulty, while “neutral”, “disagree”, and “strongly disagree” were considered as not indicating its presence.

Following the descriptive analysis of the collected data, a multilevel logistic regression was performed to explore associations between perceived barriers to pediatric research and sociodemographic characteristics. Each model included a single outcome corresponding to a specific perceived barrier or difficulty (e.g., difficulties in balancing clinical and research activities, inadequate research training, limited time for reading or writing, difficulties in data analysis, and inadequate equipment). All models included the following variables as predictors: age, sex and professional role. Region (defined as the 20 Italian administrative regions) was included as a random effect to account for clustering of responses within geographic areas, reflecting shared organizational and healthcare system characteristics. Analyses were conducted using complete-case data, as the proportion of missing values was low. No imputation procedures were applied.

Data analysis was performed using Stata 18 software (Stata Corporation, College Station, TX, USA).

## Results

A total of 887 pediatricians accessed the online questionnaire. Of these, 10 completed only the sociodemographic section and 160 were not involved in clinical and/or research activity. Thus, 717 responses were included in the analysis. The general characteristics of the respondents are presented in Table 1. The majority of respondents (97.1%) were directly involved in clinical activities, while 53.7% were actively engaged in scientific research projects. Of those involved in research, 364 (94.5%), corresponding to 50.8% of the total sample, were also involved in clinical practice, whereas only 21 pediatricians not working in clinical settings reported participation in research projects (see Additional file 1, Fig. 1). A stratified analysis by type of pediatrician was also performed, revealing differences among groups, particularly in age distribution (see Additional file 1, Table 1).

**Table 1:** Characteristics of the participants (*n* = 717)

	*n*	%
Females	489	68.2
Age (years)		
≤ 40	169	23.6
41–60	307	42.8
60+	241	33.6
Geographic area		
Northern Italy	337	47.0
Central Italy	171	23.9
Southern Italy	209	29.1
Professional category		
Family pediatrician	169	23.6
Hospital pediatrician	409	57.0
University pediatrician/Resident/PhD	139	19.4
Directly involved in clinical activities	696	97.1
Active involvement in scientific research projects	385	53.7

**Fig. 1:**
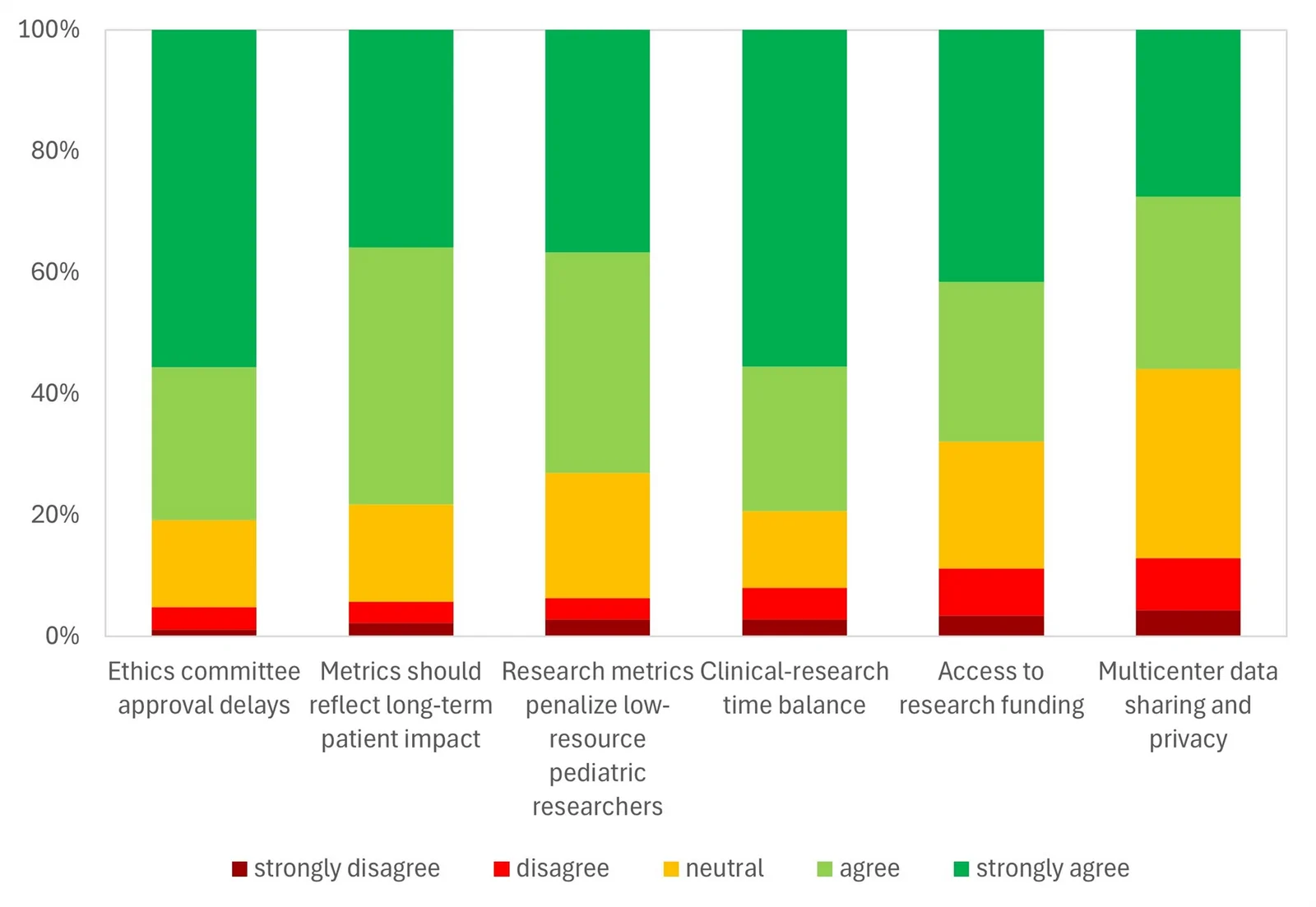
Assessment of key structural barriers and evaluation metrics in pediatric research

Among pediatricians involved in research activities (*n* = 385), the most frequently reported personal barriers to conducting research were difficulties in balancing clinical and research responsibilities and limited access to research funding (79.3% and 67.9% respectively). Challenges related to recruiting qualified research personnel (59.9%) and the availability of adequate equipment (58.7%) were also commonly reported. In contrast, work–life balance difficulties (52.6%) and inadequate research training (48.1%) were perceived as less prominent obstacles.

Pediatricians also identified objective problems that should be addressed to strengthen pediatric research. Delays in Ethics Committee approvals emerged as the most critical issue (80.8%), followed by difficulties in multicenter data sharing under privacy regulations (55.9%). Barriers to collaborative research networks (50.3%) and insufficient institutional support for artificial intelligence (AI) tools (39.8%) were perceived as moderately relevant, whereas obtaining parental consent for clinical trial participation was considered less problematic (27.6%).

The data also indicate a widely shared perception of inadequacy in current research assessment systems.

A large proportion of pediatricians (78.2%) agreed that research metrics should reflect long-term patient impact, and 78.8% supported the need to adapt evaluation metrics to the specific characteristics of pediatric research. Furthermore, 73.1% believed that current metrics penalize pediatric researchers working in low-resource contexts, while 67.3% agreed on the need to reform academic incentives beyond purely quantitative indicators. These findings are visually summarized in Fig. 1.

Among the 696 pediatricians involved in clinical activities, clinical guidelines represented the primary resource used to support clinical practice (82.3%). Systematic reviews were consulted by 55.9% of participants, while 43.5% relied on individual scientific articles. Continuing medical education (CME) courses were less frequently cited, being reported by 35.5% of respondents.

Scientific literature was perceived as having a substantial impact on clinical practice, particularly with respect to treatment effectiveness and safety: 82.8% agreed that it improves clinical outcomes, and 65.4% believed it reduces adverse events. Perceptions were more moderate for cost-effectiveness (52.6%) and especially for patient satisfaction (40.1%). Time constraints emerged as the main barrier to implementing evidence in daily practice (58.9%), followed by difficulties in assessing study quality (44.4%), applying findings to individual patients (37.7%), and interpreting results (29.3%). Limited English proficiency was considered largely irrelevant.

Regarding scientific publishing, 665 pediatricians (87.0%) reported having authored at least one scientific article.

**Fig. 2:**
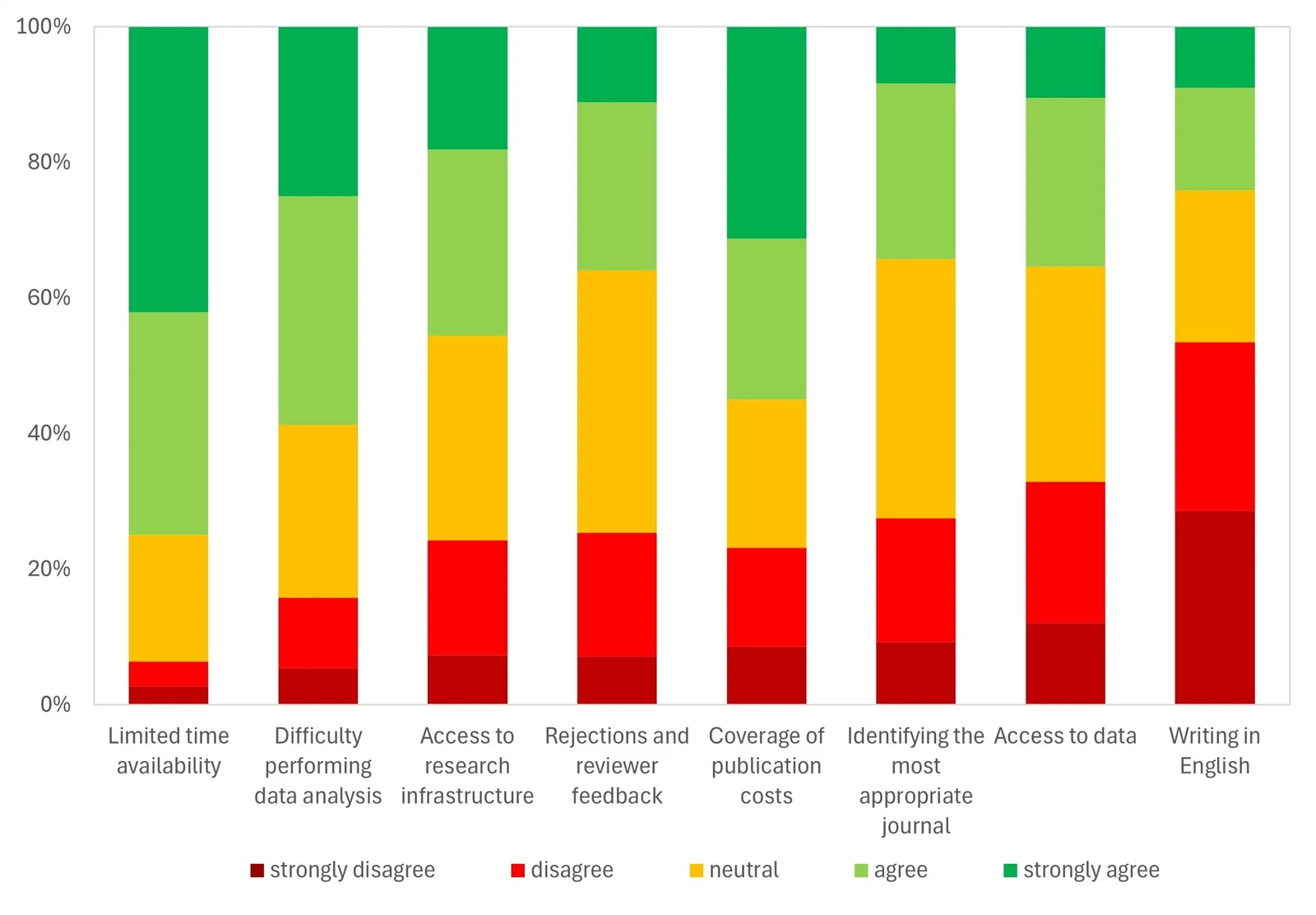
What problems do you most frequently encounter in writing and publishing your scientific articles?

Among the 665 pediatricians who reported having authored at least one scientific article, limited time was identified as a major obstacle (75%). Additional challenges included the complexity of performing statistical analyses (58.7%), limited access to research infrastructures (45.5%), and publication (55.0%). Editorial issues such as manuscript rejections and reviewer feedback (35.9%), as well as identifying an appropriate journal (34.2%), were also cited. Writing in English was not perceived as a substantial obstacle, being reported as problematic by only 24.1% of respondents (Fig. 2).

When selecting a journal, the impact factor represented the primary criterion for most participants (62.7%), followed by considerations such as the journal’s target audience and the speed of the peer-review process. In contrast, economic aspects were reported as less influential; both the absence of publication fees and open-access options ranked among the lowest priorities, with open access designated as the sixth priority by 32.7% of pediatricians.

Participants also expressed strong interest in initiatives that could support research activity and facilitate access to evidence. The most widely endorsed proposal was the creation of a monthly summary of the best pediatric research papers (88.5%). High levels of interest were also reported for training initiatives, particularly those focused on the use of AI tools (74.3%) and the critical reading of scientific literature (76.9%). High proportions of respondents expressed interest in scientific writing training (72.6%) and in bibliometric monitoring of pediatric research output (68.6%).

**Fig. 3:**
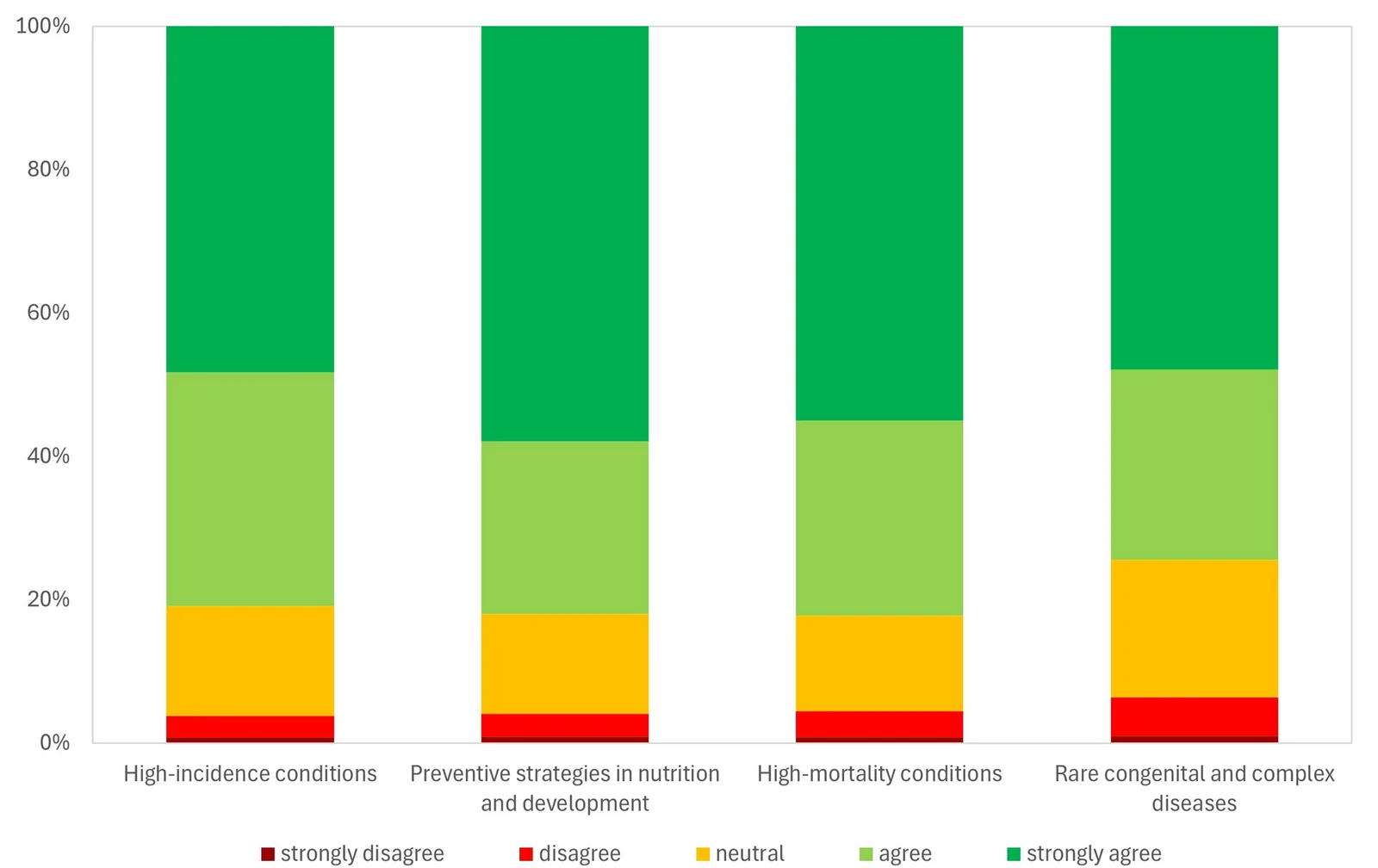
What clinical topics should be considered the most important to address in the scientific literature?

As shown in Fig. 3, among the 696 pediatricians involved in clinical activities, the diagnosis and treatment of high-incidence conditions were identified as the most important topics to be addressed in scientific literature (80.7%). Developmental preventive strategies in nutrition and neurodevelopment (76.3%) and diagnosis and treatment of high-mortality conditions (75.6%) were also highly prioritized, whereas diagnosis and treatment of rare congenital and complex diseases received slightly lower but still substantial interest (65.2%).

Finally, the multivariate analysis revealed significant differences across age groups, gender, and professional roles in relation to perceived obstacles to conducting research. Compared with pediatricians under 40 years of age, participants older than 60 were significantly less likely to report difficulties in balancing clinical work with research activities (OR = 0.29; 95% CI = 0.14–0.61; *p* = 0.001), having too little time to read the literature (OR = 0.54; 95% CI = 0.34–0.86; *p* = 0.010), or to write manuscripts (OR = 0.56; 95% CI = 0.32–0.97; *p* = 0.040). They also less frequently perceived their research training as inadequate (OR = 0.50; 95% CI = 0.28–0.89; *p* = 0.019).

Differences also emerged across professional roles: residents, PhD students, and academic pediatricians were less likely than hospital-based pediatricians to consider their research training inadequate (OR = 0.52; 95% CI = 0.32–0.87; *p* = 0.013), to report difficulties in performing data analyses (OR = 0.57; 95% CI = 0.36–0.90; *p* = 0.016), and to view inadequate equipment as a barrier (OR = 0.60; 95% CI = 0.36–1.00; *p* = 0.050). Gender differences were also observed, with women more likely than men to report difficulties in data analysis (OR = 1.53; 95% CI = 1.05–2.23; *p* = 0.027). All models are presented in Additional file 1.

## Discussion

This study provides a broad overview of Italian pediatricians’ perceptions regarding barriers and facilitators to conducting pediatric research. Many of the challenges identified align with those reported in international literature, underscoring the systemic nature of obstacles that affect pediatric research across settings.

A dominant theme emerging from our data is the lack of protected time for research. This barrier is widely recognized in both adult and pediatric clinical settings [8–10] and reflects the tension between increasing clinical demands and the need for dedicated time for scientific inquiry. High-quality research requires sustained effort for planning, data management, manuscript preparation, and collaboration. While clinical service delivery takes precedence over research, implementation of evidence based interventions is supported by engagement in clinical research [10]. Without adequate institutional support and formal recognition of research time, clinicians face a heightened risk of burnout, and the development of future pediatric investigators may be compromised. Addressing how to balance clinical workloads and research responsibilities remains a critical organizational priority.

Limited access to research funding also emerged as a major challenge. Funding constraints are well known in pediatrics, where additional ethical, logistical, and economic complexities make resource acquisition particularly demanding [11]. Strengthening political and institutional efforts to expand funding opportunities may be helpful.

Respondents also highlighted insufficient qualified personnel as a barrier, a finding congruent with previous studies [12, 13]. Effective clinical research requires data literacy, methodological expertise, and familiarity with good clinical practice, much of which can only be gained through structured training and hands-on experience. This suggests the need for strengthened academic pathways and dedicated training programs to support research competencies among pediatricians.

Participants noted that the lack of appropriate research equipment and infrastructures significantly hinders pediatric research. This issue is especially pronounced in pediatrics, where specialized, age-appropriate technologies and facilities are required, and economies of scale are inherently limited [14]. Furthermore, respondents expressed concerns about insufficient support for AI tools and indicated a strong interest in receiving training in this domain. As AI has the potential to streamline patient recruitment, enhance trial efficiency, and support clinical decision-making [15–17], investments in digital infrastructure and technology training at the local level may substantially facilitate research.

Operational obstacles were among the most frequently cited barriers. Delays in ethical approvals and challenges related to privacy management in multicenter studies were prominent concerns. Pediatric research is intrinsically susceptible to stringent ethical requirements that, while essential for patient protection, can complicate and prolong approval processes [18]. Streamlining these procedures, improving institutional support for regulatory documentation, and harmonizing processes across ethics committees could help mitigate these delays.

Bureaucracy more broadly was reported as an impediment, particularly in the context of collaborative research networks as reported in other studies [19, 20]. Complex administrative procedures required for data protection, intellectual property, and organizational agreements can slow research progress and discourage participation in multicenter initiatives. Navigating ethics committees and regulatory agencies for multicenter trials can be a labyrinthine process, consuming valuable time and resources that could be better spent on the research itself. Improving the efficiency of ethical processes through a support to preparation of complete documentation may be useful. Optimizing bureaucratic workflows and ensuring clear communication with researchers may enhance the efficiency of collaborative studies.

A notable insight from this survey is the dissatisfaction with existing criteria used to evaluate clinical research performance. Despite growing advocacy for multidimensional research metrics that capture quality, innovation, and societal impact [21–23], evaluation systems in many contexts—including Italy—remain heavily dependent on journal impact factor. This reliance shapes researchers’ priorities and may limit the pursuit of diverse research outputs.

Regarding knowledge translation, respondents identified clinical guidelines as the most important instrument for integrating research findings into practice, more so than individual articles or systematic reviews. While the relative role of different research products can be debated [24], the consensus suggests disseminating scientific knowledge through effective, accessible channels and ensuring that research outputs contribute meaningfully to patient care.

Barriers to scientific publication included limited time, difficulties in statistical analysis, and - less frequently - editorial factors such as reviewer comments or manuscript rejection. These findings parallel previous observations indicating that time constraints, fear of rejection, and limited statistical proficiency often impede manuscript submission [25, 26]. Despite recommendations for selecting journals based on scope, audience, and transparency [14], respondents predominantly considered journal impact factor as the main criterion for choosing where to publish. This preference likely reflects the strong weight currently assigned to impact factor within the Italian healthcare system’s research evaluation processes. Conversely, open access considerations ranked low among priorities.

Respondents showed similar levels of interest across the proposed research domains, with a slight preference for prioritizing diagnostic and therapeutic research in high-incidence conditions. This pattern suggests broad recognition of the need to advance evidence generation across multiple areas of pediatric care.

This study has several limitations that should be considered when interpreting the findings. First, participation was voluntary and recruitment was conducted through the communication channels of a national pediatric society (mailing list, newsletters, and social media), which may have introduced self-selection and non-response bias, potentially favoring pediatricians more interested or engaged in research. Although the society includes the large majority of pediatricians in Italy and the survey reached a substantial national sample, respondents tended to be older, and those less engaged with the society’s communications may be underrepresented, limiting generalizability. Second, the data are based on self-reported perceptions rather than objectively measured barriers, and may therefore be subject to recall bias and subjective interpretation; however, perceptions are themselves important determinants of research engagement. Third, the questionnaire was developed ad hoc and did not undergo full psychometric validation, which may affect reliability and reproducibility, although it was informed by literature review, expert input, and pilot testing, and was appropriate for the descriptive and exploratory aims of the study. In addition, the dichotomization of some Likert-scale items may have resulted in a loss of information, although this was limited to selected analyses with clear response distributions and did not affect the overall interpretation of results. Finally, the cross-sectional design captures perceptions at a single point in time and does not allow for temporal or causal inferences; therefore, the findings should be interpreted as a snapshot that may evolve over time.

In conclusion, this study highlights a complex landscape of barriers and facilitators influencing pediatric research in Italy. Key obstacles include limited protected time, insufficient funding, lack of qualified personnel, inadequate infrastructure, and burdensome regulatory and administrative processes. At the same time, pediatricians express strong motivation to engage in research and recognize the value of scientific investigation for improving clinical care. These findings suggest the need for coordinated policy actions at institutional and national levels, including strengthening organizational research support, expanding equitable funding mechanisms, investing in workforce training and digital infrastructure, and implementing regulatory reforms to streamline ethics and administrative procedures. Addressing these systemic challenges is essential not only for advancing pediatric knowledge but also for ensuring long-term improvements in population health, as today’s pediatric research forms the foundation for tomorrow’s adult well-being.

## Supplementary Information

Below is the link to the electronic supplementary material.

Supplementary Material 1

## Data Availability

The data supporting the findings of this study are available in aggregate form upon reasonable request. Requests to access data should be directed to albertoeugenio.tozzi@opbg.net.

## Abbreviations

AI: Artificial Intelligence
CME: Continuing Medical Education
NIH: National Institutes of Health

## References

[1] Bruining N, Cummins P, Serruys PW. Impact factors: scientific and career assessment by numbers. EuroIntervention. 2011;7(1):143–7. 21550915 10.4244/EIJV7I1A23

[2] Alsulami AF, Khaimi ZO, Hadi MA, Aljabri YH, Mayet TS, Althubaiti A. Publish or Perish: barriers to research publication in an undergraduate medical research program. BMC Res Notes. 2023;16(1):269. 37833749 10.1186/s13104-023-06542-5 PMC10571371

[3] Speer EM, Lee LK, Bourgeois FT, Gitterman D, Hay WW, Davis JM, et al. The state and future of pediatric research—an introductory overview. Pediatr Res. 2023;1–5. 10.1038/s41390-022-02439-4 PMC9873210 36694026

[4] Stephenson T. Importance and benefits of investing in child health and research. Glob Pediatr. 2024;9:100217.

[5] Andria G. Impact in Italy of research in pediatrics. Ital J Pediatr. 2014;40(1):A45.

[6] Corsello A, Rotulo S, Santangelo A, Diana A, Rossi F, Antonietta Catania M, et al. Challenges and opportunities in pediatric residency: an analysis of the increasing number of residents in Italy. Ital J Pediatr. 2024;50(1):206. 39380020 10.1186/s13052-024-01778-8 PMC11463055

[7] Sannino P, Maiandi S, Moretti S, Rampini S, Bonino M, Marchisio P. Bridging the gap: Barriers and opportunities in pediatric nursing research in Italy. J Pediatr Nurs. 2025;85:207–12. 40763420 10.1016/j.pedn.2025.07.034

[8] Williams J, Craig TJ, Robson D. Barriers and facilitators of clinician and researcher collaborations: a qualitative study. BMC Health Serv Res. 2020;20:1126. 33278896 10.1186/s12913-020-05978-w PMC7718701

[9] Edwards Z, Tatterton M. Barriers and facilitators to primary care staff conducting research – a qualitative systematic review. Eur J Gen Pract. 2025;31(1):2539777. 40799138 10.1080/13814788.2025.2539777 PMC12351701

[10] Drury A, Crockford C. Barriers and enablers of research engagement among multidisciplinary cancer care professionals in Ireland: A mixed-methods study. J Cancer Policy. 2025;45:100630. 40783058 10.1016/j.jcpo.2025.100630

[11] Berkley JA, Walson JL, Gray G, Russell F, Bhutta Z, Ashorn P, et al. Strengthening the paediatric clinical trial ecosystem to better inform policy and programmes. Lancet Glob Health. 2025;13(4):e732–9. 40155110 10.1016/S2214-109X(24)00511-4

[12] Harvey K, Ralph P, Spencer LH, Doungsong K, Morrison V, Lemmey A, et al. Perceived barriers and facilitators of staff recruiting participants to a randomised controlled trial of a community rehabilitation intervention following hip fracture. Trials. 2024;25(1):826. 39695752 10.1186/s13063-024-08655-z PMC11653886

[13] Briel M, Elger BS, McLennan S, Schandelmaier S, von Elm E, Satalkar P. Exploring reasons for recruitment failure in clinical trials: a qualitative study with clinical trial stakeholders in Switzerland, Germany, and Canada. Trials. 2021;22(1):844. 34823582 10.1186/s13063-021-05818-0 PMC8613940

[14] Council for International Organizations of Medical Sciences (CIOMS). In: Cobert’s manual of drug safety and pharmacovigilance [Internet]. 4a ed. WORLD SCIENTIFIC. 2025 [cited December 4, 2025]. pp. 67–86. Disponibile su: https://www.worldscientific.com/doi/10.1142/9789811297724_0006.

[15] Lin A, Wang Z, Jiang A, Chen L, Qi C, Zhu L, et al. Large language models in clinical trials: applications, technical advances, and future directions. BMC Med. 2025;23(1):563. 41088200 10.1186/s12916-025-04348-9 PMC12522288

[16] Lee K, Liu Z, Mai Y, Jun T, Ma M, Wang T, et al. Optimizing Clinical Trial Eligibility Design Using Natural Language Processing Models and Real-World Data: Algorithm Development and Validation. JMIR AI. 2024;3:e50800. 39073872 10.2196/50800 PMC11319878

[17] Lu X, Chen M, Lu Z, Shi X, Liang L. Artificial intelligence tools for optimising recruitment and retention in clinical trials: a scoping review protocol. BMJ Open. 2024;14(3):e080032. 38508642 10.1136/bmjopen-2023-080032 PMC10953313

[18] Bergstraesser E, Nadal D, Özgü H, Kleist P. Deficiencies in paediatric research applications delaying ethics committee approval. Swiss Med Wkly. 2020;150(2526):w20267–20267. 32579700 10.4414/smw.2020.20267

[19] Hertrich TJ, Brenner T. What hampers research collaboration in a region? The perspective of various stakeholders from ten German regions. Rev Reg Res. 2024;44(2):163–92.

[20] Matthews KRW, Yang E, Lewis SW, Vaidyanathan BR, Gorman M. International scientific collaborative activities and barriers to them in eight societies. Acc Res. 2020;27(8):477–95. 10.1080/08989621.2020.1774373 32515609

[21] Curry S, Gadd E, Wilsdon J. Harnessing the metric tide: indicators, infrastructures & priorities for UK responsible research assessment. [Internet]. Research on Research Institute; 2022 dic [cited December 4, 2025]. Disponibile su: https://rori.figshare.com/articles/report/Harnessing_the_Metric_Tide/21701624/2.

[22] Moher D, Bouter L, Kleinert S, Glasziou P, Sham MH, Barbour V, et al. The Hong Kong Principles for assessing researchers: Fostering research integrity. PLOS Biol. 2020;18(7):e3000737. 32673304 10.1371/journal.pbio.3000737 PMC7365391

[23] Cagan R. The San Francisco Declaration on Research Assessment. Dis Model Mech. 2013;6(4):869–70. 23690539 10.1242/dmm.012955 PMC3701204

[24] Guerra-Farfan E, Garcia-Sanchez Y, Jornet-Gibert M, Nuñez JH, Balaguer-Castro M, Madden K. Clinical practice guidelines: The good, the bad, and the ugly. Injury. 2023;54:S26–9. 35135686 10.1016/j.injury.2022.01.047

[25] Song F, Loke Y, Hooper L. Why Are Medical and Health-Related Studies Not Being Published? A Systematic Review of Reasons Given by Investigators. Wicherts JM, curatore. PLoS ONE. 2014;9(10):e110418. 25335091 10.1371/journal.pone.0110418 PMC4198242

[26] Sezer Yamanel RG, Kumru P, Kayataş Eser S, Celayir A. Barriers to writing research papers and getting them published, as perceived by Turkish physicians – a cross sectional study. Eur Sci Ed. 2021;47:e69596.

