# Supplementary material for: Perceptions of challenges in clinical pediatric research: a survey of Italian pediatricians
Source: Ital J Pediatr. 2026 Jun 12;52:129. doi: 10.1186/s13052-026-02282-x (PMC13488207; doi:10.1186/s13052-026-02282-x)
Supplement: Supplementary file 1 [file 13052_2026_2282_MOESM1_ESM.docx]

**Additional file 1**

**Questionnaire**

*Sociodemographic characteristics*

Sex (male; female; unspecified)

Age (20-30; 31-40; 41-50; 51-60; 61+)

Professional role (family pediatrician; university pediatrician; hospital pediatrician; resident-PhD)

Region

*Topic: Research*

Are you directly involved in clinical activities? (no; yes)

Are you actively involved in scientific research projects? (no; yes)

What are the main personal barriers you encounter in your work as a researcher?

Balancing the time dedicated to clinical care with the time needed for research

Difficulty balancing personal life with career

Inadequate research training

Access to research funding

Availability of adequate equipment

Difficulty recruiting qualified research personnel

What objective issues do you believe should be addressed to promote pediatric research?

The difficulty in obtaining informed consent from parents for participation in clinical studies

The difficulty and time required to obtain study approval from ethics committees

The transfer and sharing of data between institutions for multicenter studies in accordance with privacy principles

The difficulty of establishing collaborative research networks with adequate infrastructure

Support for the use of artificial intelligence tools in research activities

To what extent do you agree with the following statements?

Current research evaluation metrics disadvantage pediatric researchers working in low-resource settings

Evaluation metrics should be adapted to the specificities of pediatric research

It would be useful to develop new metrics that take into account the long-term impact of research on patients

Career-related incentives in academia should be reformed to reduce the emphasis on quantitative metrics

*Topic: Clinical application*

What types of resources do you use most frequently to guide your clinical practice? (original

articles; guidelines, systematic review; CME courses)

Which aspects, in your opinion, are most influenced by scientific literature?

Improvement of clinical outcomes

Reduction of adverse events

Patient satisfaction

Cost-effectiveness of treatments

Efficiency of care

Have you observed a measurable improvement in the quality of care for your patients after adopting evidence-based clinical practices? (often; sometimes; rarely; never; na)

Are there barriers to implementing scientific evidence in your clinical practice?

Insufficient time to review the literature

Difficulty in assessing the quality of scientific studies

Difficulty in interpreting the results of scientific studies

Uncertainty about the transferability of results to my patients

Limited proficiency in English

Which clinical topics should be prioritized in scientific literature?

Diagnosis and treatment of high-incidence conditions (diabetes, asthma, respiratory infections)

Diagnosis and treatment of conditions with higher mortality (leukemias, lymphomas, solid tumors)

Diagnosis and treatment of rare congenital disorders with high complexity

Development of prevention strategies related to nutrition and neurodevelopment

*Topic: Authorship*

Have you ever published a scientific paper, either as a sole author or together with other authors? (no; yes)

What problems do you most frequently encounter when writing and publishing your scientific articles?

Limited time available

Difficulties in performing data analysis

Access to research data

Access to research infrastructures

Covering publication fees

Manuscript rejections and responses to reviewers

Identifying the most appropriate journal

Writing in English

Which criteria do you use when choosing the journal in which to publish?

Impact Factor

Lack of peer review

Type of target readership/audience

Speed of the review process

No publication fees

Open Access

*Topic: Needs and training*

How useful would the following be:

A monthly concise update service on the most relevant pediatric research

A bibliometric monitoring service of pediatric scientific production

Training activities for the critical appraisal of scientific articles

Training activities for scientific writing

Training activities for the use of artificial intelligence tools in scientific research

Table 1. Characteristics of the participants stratified by professional category

|  | Hospital  pediatrician | | Family  pediatrician | | University pediatrician/  Resident/PhD | |
| --- | --- | --- | --- | --- | --- | --- |
|  | n | % | n | % | n | % |
| Females | 283 | 69.2 | 127 | 75.2 | 79 | 56.8 |
| Age (years) |  |  |  |  |  |  |
| ≤ 40 | 83 | 20.3 | 18 | 10.7 | 68 | 48.9 |
| 41 - 60 | 198 | 48.4 | 84 | 49.7 | 25 | 18.0 |
| 60+ | 128 | 31.3 | 67 | 39.6 | 46 | 33.1 |
| Geographic area |  |  |  |  |  |  |
| Northern Italy | 182 | 44.5 | 88 | 52.1 | 67 | 48.2 |
| Central Italy | 107 | 26.2 | 38 | 22.5 | 26 | 18.7 |
| Southern Italy | 120 | 29.3 | 43 | 25.4 | 46 | 33.1 |
| Directly involved in clinical activities | 403 | 98.5 | 164 | 97.0 | 129 | 92.8 |
| Active involvement in scientific research projects | 223 | 54.5 | 50 | 29.6 | 112 | 80.6 |

Figure 1. Graphical representation of subcategories used in the analysis

Table 2. Multilevel multivariable logistic regression analysis of factors associated with difficulties in balancing clinical work and research activities

|  | **OR** | **CI 95%** | **p** |
| --- | --- | --- | --- |
| Female | 1.65 | 0.95 - 2.85 | 0.074 |
| Age (ref: ≤ 40y) |  |  |  |
| 41 - 60 | 0.76 | 0.35 - 1.68 | 0.505 |
| 60+ | 0.29 | 0.14 - 0.61 | 0.001 |
| Professional category (ref: hospital pediatrician) |  |  |  |
| Family pediatrician | 0.61 | 0.28 - 1.34 | 0.219 |
| University pediatrician/Resident/PhD | 0.67 | 0.36 - 1.25 | 0.203 |

*Region as random effect*

Table 3. Multilevel multivariable logistic regression analysis of factors associated with view inadequate equipment as a barrier

|  | **OR** | **CI 95%** | **p** |
| --- | --- | --- | --- |
| Female | 1.27 | 0.79 - 2.03 | 0.325 |
| Age (ref: ≤ 40y) |  |  |  |
| 41 - 60 | 0.95 | 0.54 - 1.69 | 0.874 |
| 60+ | 1.31 | 0.72 - 2.40 | 0.381 |
| Professional category (ref: hospital pediatrician) |  |  |  |
| Family pediatrician | 1.67 | 0.79 - 3.56 | 0.182 |
| University pediatrician/Resident/PhD | 0.60 | 0.36 - 1.00 | 0.050 |

*Region as random effect*

Table 4. Multilevel multivariable logistic regression analysis of factors associated with perceived their research training as inadequate

|  | **OR** | **CI 95%** | **p** |
| --- | --- | --- | --- |
| Female | 1.08 | 0.69 - 1.69 | 0.727 |
| Age (ref: ≤ 40y) |  |  |  |
| 41 - 60 | 0.64 | 0.37 - 1.11 | 0.111 |
| 60+ | 0.50 | 0.28 - 0.89 | 0.019 |
| Professional category (ref: hospital pediatrician) |  |  |  |
| Family pediatrician | 0.71 | 0.36 - 1.40 | 0.329 |
| University pediatrician/Resident/PhD | 0.52 | 0.32 - 0.87 | 0.013 |

*Region as random effect*

Table 5. Multilevel multivariable logistic regression analysis of factors associated with having too little time to read the literature

|  | **OR** | **CI 95%** | **p** |
| --- | --- | --- | --- |
| Female | 1.02 | 0.70 - 1.48 | 0.927 |
| Age (ref: ≤ 40y) |  |  |  |
| 41 - 60 | 0.88 | 0.56 - 1.38 | 0.582 |
| 60+ | 0.54 | 0.34 - 0.86 | 0.010 |
| Professional category (ref: hospital pediatrician) |  |  |  |
| Family pediatrician | 1.04 | 0.69 - 1.58 | 0.850 |
| University pediatrician/Resident/PhD | 1.13 | 0.71 - 1.79 | 0.611 |

*Region as random effect*

Table 6. Multilevel multivariable logistic regression analysis of factors associated with having too little time to write manuscripts

|  | **OR** | **CI 95%** | **p** |
| --- | --- | --- | --- |
| Female | 1.33 | 0.87 - 2.04 | 0.187 |
| Age (ref: ≤ 40y) |  |  |  |
| 41 - 60 | 0.92 | 0.53 - 1.58 | 0.750 |
| 60+ | 0.56 | 0.32 - 0.97 | 0.040 |
| Professional category (ref: hospital pediatrician) |  |  |  |
| Family pediatrician | 0.68 | 0.42 - 1.11 | 0.122 |
| University pediatrician/Resident/PhD | 0.96 | 0.56 - 1.63 | 0.867 |

*Region as random effect*

Table 7. Multilevel multivariable logistic regression analysis of factors associated with having difficulties in performing data analyses

|  | **OR** | **CI 95%** | **p** |
| --- | --- | --- | --- |
| Female | 1.53 | 1.05 - 2.23 | 0.027 |
| Age (ref: ≤ 40y) |  |  |  |
| 41 - 60 | 0.80 | 0.51 - 1.27 | 0.351 |
| 60+ | 0.63 | 0.39 - 1.02 | 0.060 |
| Professional category (ref: hospital pediatrician) |  |  |  |
| Family pediatrician | 0.97 | 0.63 - 1.51 | 0.894 |
| University pediatrician/Resident/PhD | 0.57 | 0.36 - 0.90 | 0.016 |

*Region as random effect*
